# Management of moderate pain after orthopaedic surgery: A multiregional expert consensus model for postoperative and discharge protocol development

**DOI:** 10.1002/jeo2.70846

**Published:** 2026-07-13

**Authors:** Gabriele Finco, Giovanni Balato, Carlo Busatto, Gaetano Caruso, Alessandro Colosio, Antonio Coviello, Valerio Daffara, Pasquale De Negri, Alessandro Donà, Silvia Ferri, Olimpio Galasso, Giovanni Gasbarro, Federico Alberto Grassi, Massimo Innamorato, Mattia Loppini, Maria Benedetta Mascia, Anna Maria Matina, Carlo Francesco Minoli, Paolo Perna, Giacomo Placella, Elena Prisco, Laura Ramponi, Nicola Sbalzer, Pasquale Sangiovanni, Pasquale Sansone, Francesco Tasso, Andrea Tognù, Giuseppe Toro, Giulia Torsello, Alberto Di Martino

**Affiliations:** ^1^ Department of Medical Sciences and Public Health University of Cagliari Cagliari Italy; ^2^ Department of Orthopaedic Surgery and Traumatology University of Naples “Federico II” Naples Italy; ^3^ Department of Orthopaedic Surgery and Traumatology Santa Maria delle Croci Hospital Ravenna Italy; ^4^ Department of Orthopaedic Surgery and Traumatology, AOU Ferrara Arcispedale Sant'Anna University of Ferrara Ferrara Italy; ^5^ Department of Orthopaedic Surgery and Traumatology Casa di Cura Città di Parma Parma Italy; ^6^ Department of Neurosciences, Reproductive and Odontostomatological Sciences University of Naples “Federico II” Naples Italy; ^7^ Department of Orthopaedic Surgery and Traumatology Spedali Civili Brescia Italy; ^8^ Department of Anesthesia Intensive Care and Pain Medicine, AORN S.Anna & S. Sebastiano Caserta Italy; ^9^ Department of Orthopaedic Surgery and Traumatology, Sassuolo Hospital University of Modena and Reggio Emilia Sassuolo Italy; ^10^ Department of Anesthesia Intensive Care and Pain Medicine, Casa di Cura Città di Parma Parma Italy; ^11^ Department of Orthopaedic Surgery and Traumatology, AOU San Giovanni di Dio e Ruggi d'Aragona University of Salerno Fisciano Italy; ^12^ Department of Anesthesia Postoperative Intensive Care and Pain Medicine, IRCCS Rizzoli Orthopaedic Institute Argenta Ferrara Italy; ^13^ Department of Clinical, Surgical, Diagnostic and Pediatric Sciences University of Pavia Pavia Italy; ^14^ Orthopedics and Traumatology Clinic, IRCCS Policlinico San Matteo Foundation Pavia Italy; ^15^ Department of Neuroscience AUSL Romagna, Pain Unit, Santa Maria Delle Croci Hospital Ravenna Italy; ^16^ Department of Orthopaedic Surgery and Traumatology IRCCS Humanitas Research Hospital Rozzano Milano Italy; ^17^ Department of Biomedical Sciences Humanitas University Pieve Emanuele Milano Italy; ^18^ Department of Anesthesia Intensive Care and Pain Medicine, IRCCS Policlinico San Matteo Foundation Pavia Italy; ^19^ Department of Anesthesia, Intensive Care and Pain Medicine, AOU Ferrara Arcispedale Sant'Anna University of Ferrara Ferrara Italy; ^20^ UOC Week Surgery, ASST Gaetano Pini‐CTO Milan Italy; ^21^ Università Vita‐Salute San Raffaele Milan Italy; ^22^ Department of Anesthesiology, TIPO e OTI Antonio Cardarelli Hospital Naples Italy; ^23^ 1st Orthopaedic and Traumatologic Department IRCCS Istituto Ortopedico Rizzoli Bologna Italy; ^24^ Department of Anaesthesia and Intensive Care Spedali Civili di Brescia Brescia Italy; ^25^ Department of Orthopaedic Surgery and Traumatology Antonio Cardarelli Hospital Naples Italy; ^26^ Department of Women, Child and General and Specialized Surgery University of Campania “Luigi Vanvitelli” Naples Italy; ^27^ Department of Anesthesia Intensive Care and Pain Medicine, IRCCS Humanitas Research Hospital Rozzano Milano Italy; ^28^ Department of Anesthesia Intensive Care and Pain Medicine, ASST Gaetano Pini‐CTO Milan Italy; ^29^ Department of Medical and Surgical Specialties and Dentistry University of Campania “Luigi Vanvitelli” Naples Italy; ^30^ Department of Anesthesia Intensive Care and Pain Medicine, Sassuolo Hospital Italy; ^31^ Department of Biomedical and Neuromotor Science University of Bologna Bologna Italy

**Keywords:** ibuprofen, multimodal analgesia, orthopaedic surgery, paracetamol, post‐operative pain

## Abstract

Moderate post‐operative pain after orthopaedic surgery remains highly prevalent and frequently persists beyond hospital discharge, negatively impacting mobilisation, rehabilitation, patient satisfaction and risk of chronic post‐surgical pain. Despite established multimodal analgesic strategies, clinical pathways remain heterogeneous and insufficiently standardised. The objective was to develop a shared, reproducible, and implementable model for the management of moderate post‐operative pain in orthopaedic surgery, both during hospitalisation and at discharge, to support institutional protocol development. An adapted virtual Nominal Group Technique (vNGT) was used as a structured consensus methodology. Three regional expert panels were convened in Campania, Lombardia and Emilia Romagna. Before each meeting, panellists completed a structured pre‐meeting survey and received preparatory material. Each meeting followed pre‐defined vNGT stages: introduction and framing of the consensus questions, individual idea generation, structured round‐robin sharing, clarification and refinement of statements, completion of standardised decision tables and post‐meeting review/acceptance of the synthesised framework. Survey responses and decision tables were analysed qualitatively and comparatively to identify convergent principles, setting‐specific operational variables, and reproducible components. A reproducible model emerged based on systematic pain assessment, defined analgesic targets, multimodal analgesia centred on scheduled non‐opioid therapy, integration of loco‐regional techniques, rational, rescue based opioid use, structured continuity of care at discharge. Setting specific declinations were defined for: (1) Day surgery and Arthroscopy, (2) major trauma surgery in the elderly, (3) lower limb arthroplasty surgery and (4) discharge management across all settings. This vNGT‐derived framework provides an implementable structure for institutional pain management protocols in orthopaedic surgery and represents a foundation for future outcome‐based validation studies.

**Level of Evidence:**

Level N/A.

## Graphical abstract

Virtual Nominal Group Technique (VNGT)‐derived framework providing a common ground to develop institutional pain management protocols after orthopedic surgery, were developed by three regional panels of expert. Pillars of pain management included systematic pain assessment, defined analgesic targets, multimodal analgesia centered on scheduled non‐opioid therapy, integration of loco‐regional techniques, rescue based opioid use, and structured continuity of care at discharge. Specific clinical cases scenarios were provided for (1) Day surgery and Arthroscopy, (2) major trauma surgery in the elderly, (3) lower limb arthroplasty surgery and (4) discharge management across all settings.

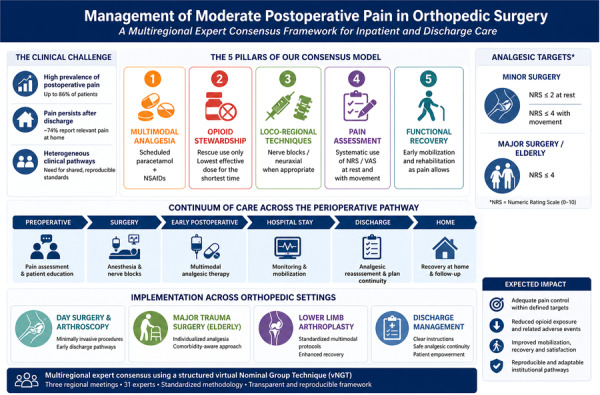

**Abbreviations**
CPSP: chronic post‐surgical pain
ERAS: Enhanced Recovery After Surgery
NRS: Numerical Rating Scale
NSAIDs: non‐steroidal anti‐inflammatory drugs
PAINAD: Pain Assessment in Advanced Dementia scale
VAS: Visual Analogue Scale
vNGT: virtual Nominal Group Technique

## INTRODUCTION

Post‐operative pain remains one of the most frequent and clinically relevant complications following orthopaedic surgery. International surveys report that up to 86% of patients experience post‐operative pain, with a substantial proportion describing moderate‐to‐severe symptoms during the early post‐operative phase [8]. Importantly, pain frequently persists after hospital discharge, negatively affecting mobilisation, rehabilitation, functional recovery, patient satisfaction, and quality of life [3, 8, 17]. Inadequate post‐operative pain control may also contribute to prolonged hospitalisation, delayed recovery, increased healthcare utilisation, and the development of chronic post‐surgical pain (CPSP), particularly after major orthopaedic procedures [9].

Over the last decade, multimodal analgesia and opioid‐sparing strategies have progressively become central components of peri‐operative care. International guidelines and Enhanced Recovery After Surgery (ERAS) pathways recommend the integration of pharmacological and procedural interventions targeting different nociceptive mechanisms in order to improve analgesia while minimising opioid exposure [4, 11]. Within this context, the combination of paracetamol and non‐steroidal anti‐inflammatory drugs (NSAIDs) has been widely adopted as a cornerstone of multimodal post‐operative pain management due to its demonstrated analgesic efficacy and opioid‐sparing effects [1, 5, 14]. At the same time, increasing awareness of opioid‐related adverse events and persistent post‐operative opioid use has reinforced the need for structured opioid stewardship strategies in orthopaedic surgery [2].

Despite the availability of evidence‐based recommendations, real‐world post‐operative pain management remains highly heterogeneous. Variability persists in pain assessment practices, peri‐operative analgesic protocols, opioid prescribing patterns, use of loco‐regional techniques, discharge planning, and continuity of care between inpatient and outpatient settings. Recent multidisciplinary consensus initiatives in orthopaedic surgery have similarly highlighted the need for standardised multimodal and opioid‐sparing peri‐operative pain management pathways [7]. This heterogeneity often limits the implementation of reproducible institutional protocols and contributes to fragmentation of post‐operative pain management across different healthcare contexts.

To address this gap, a multiregional expert‐driven initiative was conducted with the aim of developing a shared, operational, and reproducible framework for the management of moderate post‐operative pain in orthopaedic surgery, covering both hospitalisation and transition to home care. The objective of the initiative was not to generate therapeutic efficacy data, but rather to translate expert experience and evidence‐informed principles into a structured and implementable clinical framework adaptable to different orthopaedic surgical settings and regional healthcare organizations.

## METHODS

### Study design

A structured, expert‐driven consensus study was conducted using an adapted virtual Nominal Group Technique (vNGT). The vNGT was selected because it allows systematic generation, discussion, clarification, and prioritisation of expert‐derived ideas within a defined time frame, while preserving balanced participation across panellists. The methodology was adapted from established NGT procedures and from published descriptions of virtual NGT in healthcare research. The process was designed to develop an implementable framework for the management of moderate post‐operative pain in orthopaedic surgery, rather than to test the efficacy of a therapeutic intervention [6, 10, 12, 13, 15, 16].

Three regional Expert Meetings were conducted in Italy, involving Campania, Lombardia, and Emilia Romagna. Each meeting represented a separate nominal group. The use of multiple regional groups was intended to capture both common clinical principles and organizational differences across healthcare contexts. The outputs from the three groups were subsequently compared and synthesised into a single multiregional framework.

### Panel composition

The panel included 28 orthopaedic surgeons, anesthesiologists and pain specialists with direct experience in post‐operative orthopaedic pain management. Panellists were selected by the two scientific coordinators (ADM, GF) on the basis of recognised clinical expertise, professional role, and experience in the management of post‐operative pain in orthopaedic and trauma surgery. The multidisciplinary composition was intended to reflect the real‐world distribution of responsibilities across the peri‐operative pathway.

### Preparatory phase

Before each Expert Meeting, panellists received preparatory material summarising the objectives of the initiative, the clinical areas to be discussed, and the surgical scenarios to be addressed. Participants also completed a structured pre‐meeting survey exploring pain assessment practices, analgesic protocols, opioid use patterns, discharge prescriptions, perceived unmet needs and organizational barriers. Survey results were analysed in aggregated form and used to inform the discussion during each meeting. The aim of the pre‐meeting phase was to support evidence‐informed and practice‐informed idea generation, while avoiding dominance by any single participant during the synchronous discussion.

### vNGT consensus procedure

Each regional meeting followed a pre‐defined vNGT sequence. First, the scientific coordinators introduced the objectives, the clinical context, the consensus questions, and the rules of interaction. Second, participants were asked to independently reflect on the proposed surgical scenarios and to generate relevant clinical and organizational elements. Third, ideas were shared through a structured round‐robin discussion, in which each participant was invited to contribute in turn. Fourth, the proposed items were clarified, discussed, and refined to ensure shared understanding. The clarification phase was not intended to force agreement, but to ensure that each item was sufficiently explicit and clinically interpretable. Fifth, participants completed standardised decision tables for four pre‐defined scenarios: (1) day surgery and arthroscopic procedures; (2) major trauma surgery in elderly patients (3); lower limb arthroplasty surgery; and (4) discharge management across surgical settings [6, 10, 13, 15, 16].

Decision tables were structured around pre‐defined domains: responsibility, pain assessment, analgesic targets, therapeutic regimen, reassessment, and pain monitoring. The same table structure was used across the three regional meetings to ensure comparability of outputs.

### Data synthesis and consensus framework development

Survey responses and decision tables were analysed qualitatively in aggregated form. No inferential statistical comparison was planned, because the purpose of the study was consensus generation and framework development rather than hypothesis testing. The two scientific coordinators conducted a structured comparative analysis of the outputs from the three regional panels to identify: (1) recurrent and convergent clinical principles; (2) areas of regional or organizational variability; (3) setting‐specific operational requirements; and (4) reproducible components suitable for inclusion in a unified framework [12].

Items consistently emerging across the regional groups were retained as core components of the framework. Items that differed according to surgical setting, patient frailty, organizational pathway or resource availability were retained as setting‐specific declinations. The synthesised framework and the final decision tables were then circulated to the panellists for post‐meeting review and acceptance. This final review step was performed to verify clinical plausibility, completeness, and operational applicability of the consensus‐derived model.

### Ethical considerations

The initiative was based on expert discussion of clinical practice and did not involve patient‐level data collection, intervention assignment, or access to identifiable health information. Therefore, formal ethics committee approval was not required. All contributions were analysed and reported in aggregated form.

## RESULTS

### Overview of the consensus process

The structured analysis of the pre‐meeting surveys and the decision tables developed during the three Expert Meetings allowed identification of several convergent clinical principles across the participating regions. Although organizational pathways differed among the Campania, Lombardia, and Emilia Romagna healthcare systems, a consistent pattern emerged regarding the fundamental elements required for effective post‐operative pain management in orthopaedic surgery. The comparative analysis of regional practices demonstrated that the variability observed in clinical pathways primarily reflected differences in hospital organization, surgical case mix, and resource availability, rather than disagreement on clinical principles.

Across all meetings, experts converged toward a shared conceptual framework for the management of moderate post‐operative pain based on five core components:

- systematic pain assessment;
- clearly defined analgesic targets;
- scheduled multimodal non‐opioid baseline therapy;
- rational and limited opioid use;
- structured continuity of care at discharge.

This shared framework represents the conceptual backbone of the consensus‐derived model.

### Pain assessment and analgesic targets

Across all surgical settings, pain assessment was based on validated numerical scales, including the Numerical Rating Scale (NRS) or Visual Analogue Scale (VAS). Experts agreed that pain should be evaluated both at rest and during movement, with systematic documentation in medical and nursing records. Movement‐based assessment was considered particularly relevant in orthopaedic patients due to its direct relationship with functional recovery and participation in rehabilitation programmes.

The consensus‐defined analgesic targets were:

- NRS ≤ 2 at rest and ≤ 4 during movement in minor surgical procedures;
- NRS ≤ 4 in major orthopaedic surgery and elderly trauma patients.

Importantly, experts emphasised that these thresholds should not be interpreted as purely numerical targets but rather as functional indicators of adequate analgesia enabling early mobilisation and rehabilitation.

### Multimodal non‐opioid baseline therapy

A strong convergence emerged across all meetings regarding the central role of scheduled multimodal non‐opioid therapy as the cornerstone of post‐operative pain management. Experts consistently identified paracetamol and NSAIDs as the pharmacological backbone of baseline analgesia.

The rationale for this strategy lies in the complementary mechanisms of action of these agents:

- NSAIDs attenuate peripheral inflammatory sensitisation;
- paracetamol modulates central nociceptive pathways.

Randomised clinical studies have demonstrated that the combination of paracetamol and ibuprofen provides superior analgesic efficacy and safety (e.g., gastrointestinal bleeding) compared with either drug alone and significantly reduces post‐operative opioid requirements [1, 5, 14]. Fixed‐dose combinations of paracetamol (1000 mg) and ibuprofen (300 mg) were considered particularly useful for facilitating protocol adherence and reducing prescribing variability between hospital and home settings.

### Role of opioids

Experts agreed that opioids should not represent first‐line therapy for moderate post‐operative pain. Instead, opioids should be reserved for rescue therapy, to be used in:

- limited duration;
- specific clinical contexts;
- carefully selected patients.

Particular caution was recommended in elderly trauma patients due to the increased risk of opioid‐related adverse events.

### Integration of loco‐regional anaesthesia

The consensus process also highlighted the central role of loco‐regional anaesthesia techniques within multimodal analgesic strategies. Regional nerve blocks and neuraxial anaesthesia were widely recognised as effective interventions for:

- reducing early post‐operative pain intensity;
- decreasing opioid consumption;
- facilitating early mobilisation.

Although specific techniques varied among centres, their conceptual role within post‐operative analgesia pathways was consistent across regions.

### Structured discharge planning

A major point of agreement among experts concerned the importance of structured discharge planning. Postoperative pain frequently persists after hospital discharge, yet discharge analgesic prescriptions are often heterogeneous and poorly standardised.

The consensus‐derived model therefore includes several mandatory elements before discharge:

- reassessment of pain intensity;
- confirmation of analgesic targets;
- prescription of multimodal oral therapy;
- clear definition of follow‐up responsibility.

Home therapy should include a structured multimodal regimen centred on oral paracetamol and NSAIDs, with defined treatment duration and appropriate follow‐up. This structured approach aims to ensure continuity between inpatient and outpatient pain management.

### Decision tables

The consensus‐derived decision tables summarising the operational application of the model across different surgical contexts are reported as:

- Table 1 – Day surgery and arthroscopic procedures;
- Table 2 – Major trauma surgery in the elderly;
- Table 3 – Lower limb arthroplasty surgery;
- Table 4 – Discharge management across surgical settings.

**Table 1:** Post‐operative management – Day surgery and arthroscopic procedure.

Domain	Item	Description
1. Responsability	*Who*	Orthopaedic surgeon; anesthesiologist in complex cases.
2. Pain assessment	*Tools*	Numeric Rating Scale (NRS) or Visual Analogue Scale (VAS), assessed at rest and during movement/verticalization depending on procedure.
	*Frequency*	Immediately post‐operatively (return to ward/awakening/end of block), then every 6 h and at discharge.
	*Who assesses pain*	Day surgery: orthopaedic surgeon; Arthroscopic surgery with hospitalisation: ward nurse; At discharge: orthopaedic surgeon.
	*Recording*	Nursing and medical records (paper or electronic).
3. Analgesic target	*Target*	NRS ≤ 2 at rest and ≤ 4 during movement.
	*Timing*	Throughout the post‐operative period and at discharge.
	*Functional objective*	Pain should not limit prescribed post‐operative activity.
4. Therapeutic regimen	*Non‐pharmacological interventions*	Cryotherapy; compressive bandaging (when indicated); early physiotherapy (after immediate post‐operative rest). In addition: loco regional blocks and long acting neuraxial anaesthesia for arthroscopic surgery; loco regional blocks and short acting neuraxial anaesthesia with rapid motor recovery in day surgery.
	*Non‐opioid pharmacological therapy*	Paracetamol and ibuprofen fixed dose combination (1000/300 mg) as first line therapy orally every 8 hours. In surgeries associated with moderate‐severe post‐operative pain: initial IV administration (paracetamol + ibuprofen 1000/300 mg), followed by oral therapy every 8 h. Rescue therapy: ibuprofen 400 mg. If fixed dose combination is unavailable: ibuprofen 600 mg every 12 h plus paracetamol 1000 mg every 8 h until discharge. At home: continue oral combination every 8 h for 3–10 days (depending on surgery). Possible adjunct therapies include antiemetics (5‐HT3 antagonists), anti oedema agents (e.g., bromelain), and dexamethasone 8 mg in the intra and 4 mg the day after surgery.
	*Opioids*	Not routinely recommended. In selected cases (NRS ≥ 7): oxycodone controlled release 5 mg every 12 h or tapentadol 50 mg every 12 h (maximum 48 h), or tramadol oral drops (dose adjusted according to patient characteristics).
5. Therapy reassessment and pain management	*Who*	Nurse (initial evaluation); orthopaedic surgeon (first therapeutic adjustment); anesthesiologist for complex cases.
	*When*	During hospitalisation (every 6–8 h) until discharge.

**Table 2:** Post‐operative management – Major trauma surgery in the elderly.

Domain	Item	Description	
1. Responsability	*Who*	Multidisciplinary team (possible inclusion of geriatrician).	
2. Pain assessment	*Assessment tools*	Numeric Rating Scale (NRS) or Visual Analogue Scale (VAS), at rest and/or during movement/verticalization (depending on procedure), Wong–Baker Faces Scale and/or Pain Assessment in Advanced Dementia scale (PAINAD) scale in elderly patients with neurodegenerative disease or cognitive impairment.	
	*Frequency*	Immediately post‐operatively (upon return to ward/end of block), then every 6 h until discharge.	
	*Who assesses pain*	Ward nurse; integrative role of anesthesiologist (first 24 h) and orthopaedic surgeon (entire hospitalisation).	
	*Where it is recorded*	Nursing and medical records (paper or electronic).	
3. Analgesic target	*Target*	NRS ≤ 4.	
	*Timing*	Throughout post‐operative period and at discharge.	
	*Functional objective*	Early mobilisation and avoidance of prolonged bed rest; no limitation of prescribed activity due to pain.	
4. Therapeutic regimen	*Non‐pharmacological interventions*	Cryotherapy, compressive bandaging when indicated, early physiotherapy (rest in immediate post‐operative period), long acting or continuous loco regional blocks and/or neuraxial anaesthesia, prefer short acting or low concentration and/or continuous anaesthetics when possible; caregiver support.	
	*Non‐opioid pharmacological therapy*	For major surgery and when clinically appropriate (e.g., no moderate/severe renal impairment): paracetamol/ibuprofen 1000/300 mg every 12 h. For minor procedures: paracetamol 1000 mg every 8 h. The paracetamol/ibuprofen 1000/300 mg fixed dose combination should be administered intravenously during the first 24 h; subsequent administration, either oral or intravenous, should be determined based on pain intensity and the feasibility of oral intake. Duration: until discharge. Other medications: possible association with 5‐HT3 antiemetic, dexamethasone 8 mg intraoperatively and 4 mg the following day; other analgesic options: celecoxib, etoricoxib, nimesulide, diclofenac.	
	*Opioids*	As rescue: Oramorph 5 mg every 8 h or oxycodone/naloxone 5/2.5 mg orally every 12 h or tapentadol 25 mg orally every 12 h (selection based on patient profile).	
5. Therapy reassessment and pain monitoring	*Who*	Nurse (first evaluation step); orthopaedic surgeon (first additive treatment step); geriatrician/anesthesiologist for preoperative planning.	
	*When*	Throughout hospitalisation (every 6–8 h) until discharge.	

**Table 3:** Post‐operative management – Lower limb arthroplasty surgery.

Domain	Item	Description
1. Responsability	*Who*	Multidisciplinary team, with possible inclusion of a geriatrician when clinically indicated.
2. Pain assessment	*Assessment tools*	Numeric Rating Scale (NRS) or Visual Analogue Scale (VAS), assessed at rest and/or during movement/verticalization (depending on procedure); Wong–Baker Faces Scale in elderly patients with neurodegenerative disorders.
	*Frequency*	Immediately post‐operatively (upon return to ward or end of anaesthetic block), then every 6 h until discharge.
	*Who assesses pain*	Nurse and orthopaedic surgeon; anesthesiologist on call for selected cases.
	*Where it is recorded*	Nursing and medical records (paper or electronic).
3. Analgesic target	*Target*	NRS ≤ 4 during movement.
	*Timing*	Throughout the post‐operative period and at discharge.
	*Functional objective*	Early mobilisation; avoidance of prolonged bed rest; no limitation of prescribed activity (including physiotherapy) due to pain.
4. Therapeutic regimen	*Non‐pharmacological interventions*	Cryotherapy; compressive bandaging when indicated; early physiotherapy (with rest in the immediate post‐operative phase); long acting or continuous loco regional blocks and/or neuraxial anaesthesia.
	*Non‐opioid pharmacological therapy*	The paracetamol/ibuprofen 1000/300 mg fixed dose combination every 8 h (IV during first 24–48 h, then IV or oral according to pain intensity); possible association with 5‐HT3 antiemetics and dexamethasone 8–12 mg perioperatively.
	*Opioids*	Continuous IV morphine infusion or low dose neuraxial morphine perioperatively; intrathecal sufentanyl; continuous IV tramadol infusion with metoclopramide during first 24–48 h post‐surgery.
	*Rescue therapy*	Oramorph 5 mg every 8 h or oxycodone/naloxone 5/2.5 mg orally every 12 h or tapentadol 25 mg orally every 12 h (based on patient profile).
5. Therapy reassessment and pain monitoring	*Who*	Nurse (first evaluation step); orthopaedic surgeon (first therapeutic adjustment); anesthesiologist (second‐line escalation).
	*When*	Continuous monitoring during hospitalisation; reassessment every 6–8 h until discharge.

**Table 4:** Discharge – All surgical settings.

Domain	Item	Description
1. Responsability	*Who*	Primarily orthopaedic surgeon, with anesthesiology or multidisciplinary support in complex patients.
2. Pain assessment	*Assessment tools*	Numeric Rating Scale (NRS) or Visual Analogue Scale (VAS), assessed at rest and/or during movement/verticalization (depending on procedure).
	*Frequency*	At discharge.
	*Who assesses pain*	Orthopaedic surgeon.
	*Where it is recorded*	Nursing and medical records (paper or electronic).
3. Analgesic target	*Target*	NRS ≤ 4 during movement.
	*Timing*	At discharge.
	*Functional objective*	Progressive functional autonomy and participation in rehabilitation without pain related limitation.
4. Therapeutic regimen	*Non‐pharmacological interventions*	Cryotherapy; compressive bandaging when indicated; physiotherapy; adequate caregiver support.
	*Non‐opioid pharmacological therapy*	Oral fixed dose combination of paracetamol + NSAID (e.g., paracetamol/ibuprofen 1000/300 mg) every 8 hours, for up to 10 days; alternative NSAIDs include celecoxib, etoricoxib, nimesulide, diclofenac, and ketoprofen.
	*Opioids*	Not routinely recommended; in selected cases: tapentadol 50 mg orally every 12 h.
		Treatment must be individualised according to patient condition and type of surgery.
5. Therapy reassessment and pain monitoring	*Who*	General practitioner or healthcare facility (nursing home, long‐term care, rehabilitation); emergency department or discharging ward in case of urgency; orthopaedic surgeon at follow up visit.
	*When*	Reassessment as clinically indicated and at scheduled follow up.

**Figure 1:**
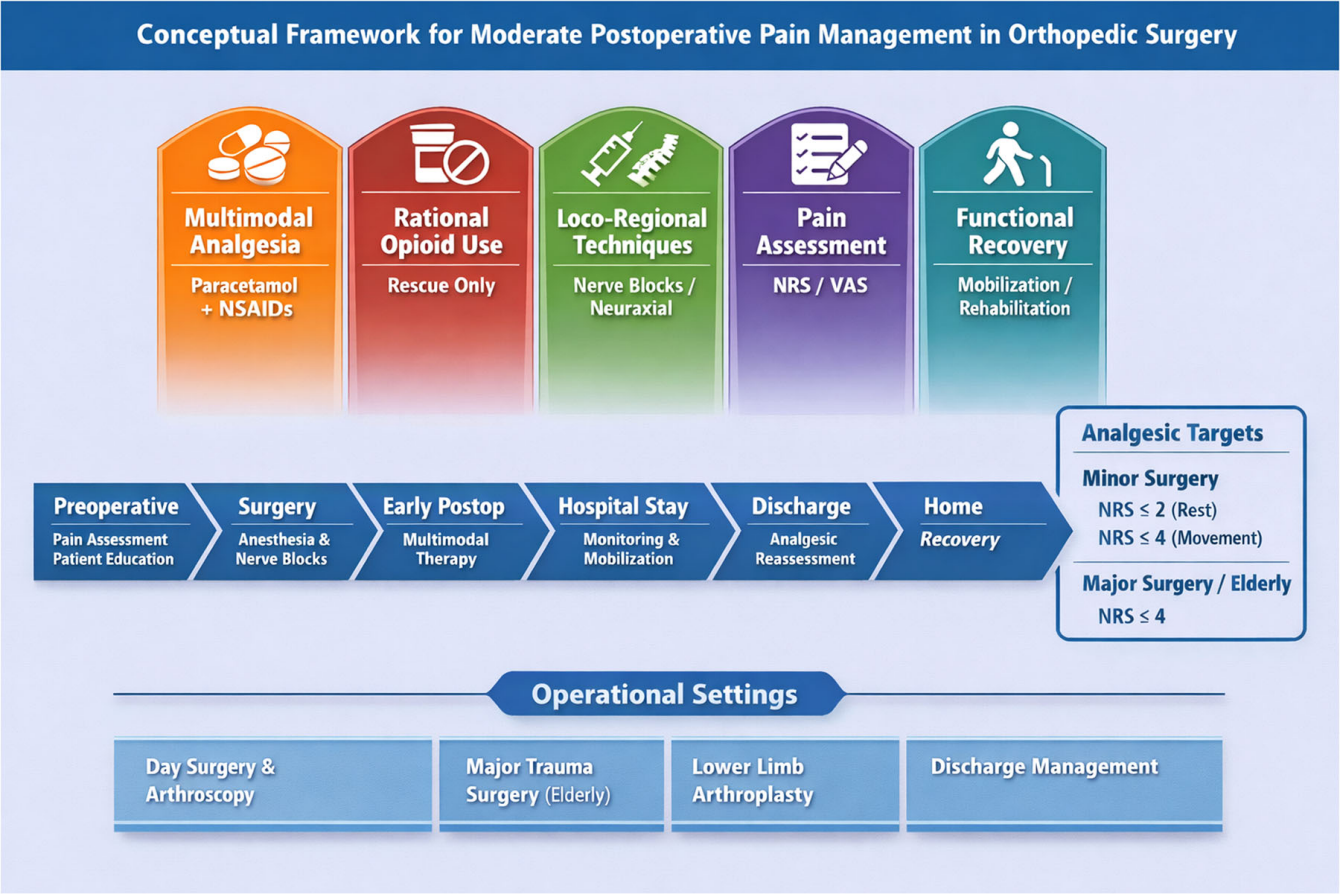
Conceptual framework for the management of moderate post‐operative pain in orthopaedic surgery. The model illustrates the continuum of care from the preoperative phase to home recovery. Multimodal analgesia centred on non‐opioid baseline therapy represents the pharmacological backbone of the strategy, supported by systematic pain assessment, rational opioid stewardship, and integration of loco‐regional anaesthesia techniques. The framework is operationalized across different surgical settings through structured decision tables.

These tables represent the operational translation of the shared framework identified through the comparative analysis of regional expert practices. The key messages are also resumed in Figure 1.

## DISCUSSION

The present work proposes a shared and structured framework for the management of moderate post‐operative pain in orthopaedic surgery, developed through a multiregional expert‐driven consensus process. Unlike purely theoretical models, the framework presented here derives from the structured comparison of real‐world clinical practices across different regional healthcare systems. The decision tables developed during the Expert Meetings allowed the translation of clinical experience into operational pathways, which were subsequently analysed and synthesised into a unified framework. Despite differences in organizational structures, hospital resources, and local prescribing habits, experts consistently converged toward a common set of clinical principles guiding post‐operative pain management.

These principles include:

- systematic pain assessment;
- clearly defined analgesic targets;
- multimodal baseline therapy centred on non‐opioid agents;
- rational opioid stewardship;
- integration of loco‐regional anaesthesia techniques;
- structured discharge planning.

This convergence suggests that variability observed in clinical practice is less related to disagreement on clinical principles and more to the absence of structured frameworks capable of organising those principles into reproducible clinical pathways.

### Multimodal analgesia as the cornerstone of post‐operative pain management

A central finding of the consensus process was the unanimous recognition of multimodal analgesia as the fundamental strategy for post‐operative pain control. These findings are consistent with recent Italian Delphi consensus recommendations emphasising multimodal and multidisciplinary post‐operative pain management strategies in orthopaedic and trauma surgery [7]. Post‐operative orthopaedic pain is characterised by a complex pathophysiology involving inflammatory activation, peripheral nociceptor sensitisation, central pain modulation, and mechanical stimulation associated with mobilisation and rehabilitation. For this reason, single‐agent analgesic approaches are often insufficient.

International guidelines consistently recommend multimodal analgesia as the standard of care for post‐operative pain management [4]. Multimodal analgesia should be interpreted not merely as the combination of multiple drugs, but as a strategic integration of pharmacological and procedural interventions acting at different levels of the nociceptive pathway. This approach has been shown to improve pain control, reduce opioid consumption, accelerate functional recovery, decrease post‐operative complications.

In orthopaedic surgery, multimodal analgesia is also a key component of ERAS pathways, which emphasise early mobilisation and optimized peri‐operative care [11].

### Role of non‐opioid baseline therapy

Another major point of agreement among experts concerned the central role of scheduled non‐opioid baseline therapy. Paracetamol and NSAIDs represent the pharmacological backbone of multimodal post‐operative analgesia. These drugs act through complementary mechanisms:

- NSAIDs reduce peripheral inflammatory sensitisation by inhibiting prostaglandin synthesis;
- paracetamol acts primarily through central mechanisms involving serotonergic and endocannabinoid pathways.

Randomised clinical trials have demonstrated that the combination of paracetamol and ibuprofen provides superior analgesic efficacy and safety (e.g., gastrointestinal bleeding) compared with monotherapy and significantly reduces post‐operative opioid requirements [1, 5, 14]. The opioid‐sparing effect of multimodal non‐opioid therapy is particularly relevant in orthopaedic surgery, where early mobilisation and rehabilitation are critical determinants of functional recovery. Fixed‐dose combinations of paracetamol and ibuprofen may further facilitate the implementation of multimodal analgesia by simplifying prescribing patterns and improving adherence to clinical protocols. In addition, reducing NSAIDs dose albeit maintaining efficacy, the fixed‐dose combinations have better safety profile. The availability of both intravenous and oral formulations also allows continuity between the early post‐operative phase and home management.

### Opioid stewardship in post‐operative orthopaedic pain

The present framework explicitly adopts an opioid stewardship approach. Although opioids remain essential for the management of severe acute pain, their routine use for moderate post‐operative pain is increasingly questioned. Evidence suggests that early post‐operative opioid exposure may increase the risk of persistent opioid use [2]. Furthermore, opioid‐related adverse effects, such as nausea, vomiting, sedation, respiratory depression, delirium, may significantly complicate post‐operative recovery, particularly in elderly trauma patients. For this reason, the consensus model recommends reserving opioids for rescue therapy rather than routine baseline treatment. This approach aligns with contemporary opioid‐sparing strategies promoted by international peri‐operative guidelines and recent national multidisciplinary consensus recommendations [7].

### Integration of loco‐regional anaesthesia

Another key pillar of the proposed framework is the integration of loco‐regional anaesthesia techniques. Regional nerve blocks and neuraxial anaesthesia have been shown to significantly reduce early post‐operative pain, decrease opioid requirements, facilitate early mobilisation, and improve patient satisfaction.

These techniques are widely recommended in orthopaedic surgery, particularly in arthroplasty and trauma procedures [4, 11]. The present consensus process confirmed their central role within multimodal analgesia pathways. Importantly, the framework does not consider loco‐regional anaesthesia as an isolated intervention but integrates it within a broader peri‐operative strategy consistent with ERAS principles.

### Continuum of care across the peri‐operative trajectory

A particularly relevant conceptual contribution of the present model is the explicit recognition of pain management as a continuum of care extending across the entire peri‐operative trajectory. Within ERAS pathways, pain assessment and analgesic planning should begin at the first surgical consultation and continue through hospitalisation and discharge to home recovery. Early patient engagement allows clinicians to anticipate post‐operative analgesic needs, initiate baseline therapy when appropriate, and educate patients on post‐operative pain expectations and management strategies.

Early and adequate analgesia also plays a crucial role in the prevention of chronic post‐surgical pain. Severe uncontrolled acute pain has been identified as one of the main predictors of chronic post‐surgical pain, which may develop in up to 10%–30% of surgical patients [9]. By promoting early multimodal analgesia and minimising unnecessary opioid exposure, the proposed framework aligns acute pain management with long‐term preventive objectives.

### Importance of structured discharge planning

Perhaps the most innovative dimension of the present work is the explicit inclusion of the discharge phase as a structured component of the analgesic pathway. Post‐discharge pain remains highly prevalent, with many patients continuing to experience moderate pain after returning home [3, 8]. However, discharge prescriptions are often heterogeneous and poorly standardised. The consensus model therefore introduces several mandatory elements before discharge:

- reassessment of pain intensity;
- verification of analgesic targets;
- definition of oral multimodal therapy;
- assignment of follow‐up responsibility.

By extending the analgesic strategy beyond hospitalisation, the framework addresses one of the most important but often overlooked contributors to persistent post‐operative pain and inappropriate opioid continuation. The use of fixed‐dose paracetamol/ibuprofen combinations across both inpatient and discharge phases further supports continuity of care and reduces variability in prescribing patterns.

### Organizational implications

Beyond pharmacological aspects, the framework also has relevant organizational implications. By clearly defining roles among orthopaedic surgeons, anesthesiologists, pain specialists, and primary care physicians, post‐operative pain management becomes a structured multidisciplinary process rather than an episodic intervention. This approach may facilitate institutional protocol development, quality improvement initiatives, benchmarking across centres.

### Limitations

Several limitations should nevertheless be acknowledged. The present framework derives from expert consensus rather than prospective randomised trials. Although grounded in current evidence and guideline recommendations, prospective validation studies will be necessary to evaluate its impact on clinical outcomes such as post‐operative pain control, opioid consumption, rehabilitation outcomes, and incidence of chronic post‐surgical pain.

## CONCLUSIONS

In conclusion, this work demonstrates that a flexible yet standardised framework for moderate post‐operative orthopaedic pain management is both achievable and reproducible across diverse clinical settings. By combining multimodal pharmacology, loco regional techniques, opioid stewardship, and structured continuity of care, the model provides a pragmatic foundation for institutional protocol development and future clinical validation.

## AUTHOR CONTRIBUTIONS

Gabriele Finco and Alberto Di Martino conceived the review, wrote the paper and supervised the final results. All authors contributed to the development of the results through participation at the VNGT process, development and revision of protocols, and revised and improved the paper up to its final form.

## FUNDING INFORMATION

Angelini Pharma.

## CONFLICT OF INTEREST STATEMENT

G.F. has received speaker fees from several pharmaceutical companies (including Janssen, Grünenthal, Pfizer, Sandoz, Abbott, Alfa Wassermann, Mundipharma, Boehringer Ingelheim, Chiesi, GlaxoSmithKline, IBSA, Mylan, Agave, Menarini, Angelini, and Zambon) and research funding from Janssen, Grünenthal, Pfizer, Fondazione Banco di Sardegna, PNRR 2023, and PRIN 2022. All the authors declare a conflict of interest with Angelini related to the present work.

## ETHICS STATEMENT

The authors have nothing to report.

## Supporting information


**Table S1.** Structured pre meeting survey.

## Data Availability

The authors have nothing to report.
