# Supplementary material for: Management of moderate pain after orthopaedic surgery: A multiregional expert consensus model for postoperative and discharge protocol development
Source: J Exp Orthop. 2026 Jul 13;13(3):e70846. doi: 10.1002/jeo2.70846 (PMC13361057; doi:10.1002/jeo2.70846)
Supplement: Supplementary file 1 — Table S1. Structured pre meeting survey. [file JEO2-13-e70846-s001.docx]

| **Question** | **Answers** |
| --- | --- |
| *Is there a protocol in your center for the management of pain at discharge following orthopedic surgery?* | - Yes - No |
| *Who is responsible for the therapeutic decision at the time of discharge?* | - Orthopedic surgeon - Multidisciplinary team - Anesthesiologist / pain specialist - Other |
| *Is pain assessed at the time of discharge?* | - Yes - No - If the patient reports pain - Other |
| *Which analgesics are most frequently used at discharge for moderate pain (oral administration)? (Multiple answers possible)* | - NSAIDs - Paracetamol - Weak opioids - Combinations |
| *What dosing regimen is followed for administration at discharge?* | - Around the clock (scheduled dosing) - As needed - Other |
| *H ow are the two active ingredients, paracetamol and ibuprofen, used in your center at discharge following orthopedic surgery? (Multiple answers possible)* | - Together, but in separate formulations - As a fixed-dose combination - In alternation - In combination with other analgesics |
| *In which types of procedures would you consider the fixed-dose combination of paracetamol 1000 mg + ibuprofen 300 mg (oral solution) to be most appropriate? (Multiple answers possible)* | - Minor/moderately invasive surgery (e.g., arthroscopy, ligament reconstruction) - Early discharge setting or day surgery - Trauma surgery (fractures requiring reduction and fixation) - Major orthopedic surgery (e.g., hip or knee replacement) |
| *At which stage of the surgical pathway would you consider it a priority to introduce the combination to maximize its effectiveness in managing moderate pain? (Select one answer, considering all types of surgeries performed in your center)* | - At discharge - Immediate postoperative period (first 48 hours) - Preoperative phase - Only if pain persists at discharge - Other |
| *Main barriers to the adoption of the combination. (Multiple answers possible)* | - Cultural resistance / prescribing habits - Lack of institutional (hospital) protocols - Safety concerns or drug interactions in specific patient populations (renal, gastrointestinal, cardiovascular comorbidities) - Lack of clinical data - Other |
| *Key drivers (advantages) to facilitate the adoption of the combination. (Multiple answers possible)* | - Improved adherence and home management - Reduction in opioid use - Better pain control in the first 48 hours - Other |
| *Please indicate one priority for improvement in your center regarding the management of moderate pain at discharge following orthopedic surgery. (Open-ended response)* | - |
